# Atorvastatin Inhibits Breast Cancer Cells by Downregulating PTEN/AKT Pathway via Promoting Ras Homolog Family Member B (RhoB)

**DOI:** 10.1155/2019/3235021

**Published:** 2019-03-18

**Authors:** Qing Ma, Yang Gao, Pei Xu, Kai Li, Xiaolong Xu, Jingbo Gao, Yuwen Qi, Jingjing Xu, Yan Yang, Wenjing Song, Xin He, Shuting Liu, Xiaoning Yuan, Weinan Yin, Yanqi He, Wenting Pan, Lei Wei, Jingwei Zhang

**Affiliations:** ^1^Department of Breast and Thyroid Surgery, Zhongnan Hospital, Hubei Key Laboratory of Tumor Biological Behaviors, Hubei Cancer Clinical Study Center, Wuhan University, Wuhan 430071, Hubei, China; ^2^Department of Pathology and Pathophysiology, Hubei Provincial Key Laboratory of Developmentally Originated Disease, School of Basic Medical Sciences, Wuhan University, Wuhan 430071, Hubei, China; ^3^Department of Cardiothoracic Surgery, Xinhua Hospital, School of medicine, Shanghai Jiao Tong University, Shanghai 200092, China

## Abstract

**Background:**

Breast cancer (BC) is one of the most common malignant tumors in women around the world. Atorvastatin (ATO) was found to be associated with a decreased risk of recurrence and mortality in cancer. But the exact mechanism of its carcinostatic effects is unclear. The expression level of Ras homolog family member B (RhoB) in breast cancer cells was found to be upregulated after being treated with ATO. Thus, we conjecture that altered expression of RhoB induced by ATO may be decisive for the migration and progression of breast cancer.

**Methods:**

The effects of ATO on breast tumor cells* in vivo* and* in vitro* were detected by clone formation assay, CCK-8 assay, flow cytometry, wound healing, transwell assays, tumor xenograft model, and immunohistochemistry. Distribution of RhoB in different breast cancer tissues and its influence on prognosis were analyzed using the data from TCGA or GEO databases. The relationship between RhoB and PTEN/AKT pathway was detected by Western blotting and RT-qPCR.

**Results:**

ATO inhibits proliferation, invasion, EMT, and PTEN/AKT pathway and promotes apoptosis in breast tumor cells. In addition, ATO inhibits the volume and weight of breast tumor in tumor-bearing mice and upregulated RhoB in tumor tissues. The expression of RhoB in mRNA and protein level was upregulated in statin-treated breast cancer cells and downregulated in cancer tissues. Low expression of RhoB links with poor prognosis in patients with breast cancer (HR = 0.74[0.66–0.83],* p* =7e^−8^, log-rank test). Further research found that RhoB inhibits the proliferation, invasion, EMT, and PTEN/AKT signal pathway in breast tumor cells.

**Conclusions:**

The exact mechanism of ATO's carcinostatic effects in breast cancer is related to downregulating PTEN/AKT pathway via promoting RhoB. Our study also demonstrates the potential applicability of RhoB as a therapeutic target for breast cancer.

## 1. Introduction

Among women worldwide, breast cancer is the leading cause of death and the most common type of solid tumor [1]. Currently, treatments for breast cancer are surgery, radiotherapy, hormone therapy, adjuvant chemotherapy, and targeted therapy [2]. However, because of the heterogeneity of breast cancer, some patients do not respond to above-mentioned treatments. Therefore, developing new therapies is paramount to decrease breast cancer related mortality and improve overall survival [2].

Women with high cholesterol have a higher incidence of breast cancer [3]. The mevalonate pathway serves as the vital pathway for the production of cholesterol [3]. Products of mevalonate pathway have been reported to promote migration, proliferation, differentiation, and intracellular trafficking of tumor cells [4]. For instance, isoprenoid promotes Ras and Rho GTPase prenylation [5], which activates the PI3K/AKT pathway and contributes to the development of tumorigenesis [6]. Thus, inhibiting the mevalonate pathway via statins may have significant inhibitory influences on cancer cell growth [7, 8].

Atorvastatin (ATO) is a statin that inhibits the function of the rate-limiting enzyme 3-hydroxy-3-methylglutaryl-CoA (HMG-CoA) reductase. ATO has been widely used to lower lipid levels and reduce cardiovascular risk [9]. Nowadays, ATO was found to be associated with a decreased risk of recurrence and mortality in cancer [10, 11]. Previous animal studies have found that ATO effectively inhibits tumor growth in breast, prostate, pancreatic, and liver cancer [12–14]. Moreover, ATO shows antiproliferative effects on different cancer cells including breast cancer cells. Therefore, ATO has gained increased interest as a potential therapeutic agent for use as an anticancer treatment [15]. Although the exact mechanism of its carcinostatic effects is currently unknown, ATO both modifies the cell cycle and induces growth suppression or apoptosis of malignant cells.

A window-of-opportunity phase II trial revealed that 21 genes were upregulated including RhoB in breast cancer tissues after the patients treated with ATO [16]. As a member of the Ras superfamily of isoprenylated small GTPases, RhoB has the function of regulating actin stress fibers and vesicle trafficking [17]. RhoB usually acts as a tumor suppressor gene because it inhibits tumor cell proliferation and migration and promotes apoptosis of tumor cells by inhibiting PTEN/AKT pathway [18]. Thus, we conjecture that altered expression of RhoB induced by ATO might be decisive for breast cancer migration and progression.

In this study, we intend to study the inhibitory effects of ATO on breast cancer cells and related mechanisms and secondly to evaluate the role of RhoB in breast cancer. Furthermore, in breast cancer cells, we initially determined that ATO inhibits PTEN/AKT signaling pathway by upregulating RhoB. Our findings complement the mechanism by which ATO inhibits breast cancer and demonstrates the potential of RhoB to become a biomarker for breast cancer.

## 2. Materials and Methods

### 2.1. Cell Culture and Drug Treatment

Human breast cancer cell lines MDA-MB-231 and MCF-2 were obtained from the China Center for Type Culture Collection (Shanghai, China). MCF-7 was cultured in MEM medium (HyClone, USA) containing 10% fetal bovine serum (FBS, Gibco, Milano, Italy), 1% Penicillin-Streptomycin Solution (HyClone, USA), and 10mg/mL insulin solution (Sigma-Aldrich, USA) and incubated in a humidified incubator at 37°C, with 5% CO_2_. MDA-MB-231 was incubated in L-15 medium (HyClone, USA) supplemented with the same concentration of FBS and Penicillin-Streptomycin Solution without CO_2_.

The cells were treated with atorvastatin calcium (C_66_H_68_CaF_2_N_4_O_10_, Solarbio, Beijing, China). ATO was dissolved in dimethyl sulfoxide (DMSO, Sigma-Aldrich, USA) and stored at 4°C.

### 2.2. Human Breast Cancer Samples and Antibodies

We obtained three cases of breast cancer and paired paracancerous from Zhongnan Hospital of Wuhan University. These tissues are diagnosed by the Department of Pathology. Before the operation, the patients were informed and signed the informed consent form. The study was endorsed by the Ethics Committee of the Zhongnan Hospital of Wuhan University and was conducted in accordance with all relevant principles of the Helsinki Declaration. The primary antibodies used in immunohistochemistry and Western blotting are as follows: RhoB (Proteintech, USA), PTEN (Proteintech, USA), AKT (Proteintech, USA), p-AKT (Proteintech, USA), E-cadherin (Cell Signaling Technology, USA), snail (Proteintech, USA), vimentin (Cell Signaling Technology, USA), *β*-actin (Proteintech, USA), and GAPDH (Proteintech, USA).

### 2.3. Plasmids, siRNA, and Cell Transfections

The RhoB gene (NM_004040.3) was amplified by PCR from genomic DNA of MCF-7 cells as a template. The primers are listed below: sense: 5'-CGGAATTCCATGGCGGCCATCCGCAAGAA-3', antisense: 5'-CGGGATCCTCATAGCACCTTGCAGCAGT-3'. PCR product (590 bp) was digested with EcorI/BamHI and ligated into pflag-CMV vector (Clontech Laboratories Inc.) to yield flag-RhoB which was confirmed by DNA sequencing.

The 21-nucleotide siRNA was produced by GenePharma (Shanghai, China) in sense and antisense directions according to human RhoB. The sequences of si-RhoBs are listed below: si-RhoB1 (sense: 5'-CCGUCUUCGAGAACUAUGUTT-3' and antisense: 5'-ACAUAGUUCUCGAAGACGGTT-3'), si-RhoB2 (sense:5'-GCUGAUCGUGUUCAGUAAGTT-3' and antisense: 5'- CUUACUGAACACGAUCAGCTT-3'), and si-RhoB3 (sense: 5'-ACGUCAUUCUCAUGUGCUUTT-3' and antisense: 5'-AAGCACAUGAGAAUGACGUTT-3'). A negative siRNA control (sense 5'-UUCUCCGAACGUGUCACGU-3' and antisense 5'-ACGUGACACGUUCGGAGAA-3') was purchased meanwhile.

Breast cancer cells were incubated in six-well plates (5×10^5^ cells per well), until they reached 70% density after 24 h. For overexpression, MDA-MB-231 cells were transfected with flag-RhoB or flag-NC using Lipofectamine 2000 reagent (Invitrogen Co., Ltd.). Diluted liposome (8 *μ*L per well) was mixed with plasmid (4 *μ*g per well) in MEM sans FBS and antibiotics. For knockdown, MCF-7 cells were transfected with siRNAs or negative control. For each well, the transfection complex containing 100 pmol diluted siRNAs and 5 *μ*L diluted liposome. Transfection mixture was incubated at room temperature for 25 minutes and then added to each well containing cells with 2 ml medium. After transfection 48 h, the cells were analyzed by RT-qPCR and Western blotting.

### 2.4. Data Collection and Analysis

The transcriptome profiling data GSE33552 was downloaded from GEO (Gene Expression Omnibus) database in the NCBI, which was deposited by Vintonenko N et al. [19]. The datasets include expression data from MDA-MB-231 cell line treated with Zoledronate or Fluvastatin and mock-treated control cells. The differential expression genes were chosen with restricted filtration (Fold Change ≥10). UCSU Xena (https://xenabrowser.net/datapages/) was used to analyze the mRNA expression of RhoB in breast cancer tissues or normal breast tissues and the clinical data of the corresponding patients in the TCGA database. The content of RhoB mRNA was normalized. The difference of RhoB mRNA expression between 1247 samples was compared according to PAM50 subtype and ER (Estrogen Receptor) status. Kaplan-Meier Plotter [20] was used to draw survival curves to compare the overall survival time in 3951 patients who have been grouped by levels of RhoB expression.

### 2.5. Cell Proliferation Assay

We performed CCK-8 and clone formation assays to detect the proliferative capacity of breast cancer cells. For the CCK-8 assay (Dojindo Laboratories, Japen), cells were seeded at a density of 1000 cells per well in 96-well plates and incubated overnight, then cells in logarithmic growth phase were treated with different concentrations of ATO ranging from 0.5 *μ*M to 32 *μ*M for 48 h or 72 h. For groups other than the NC group, the same quantity of DMSO (5 *μ*M) with different concentrations of ATO was used to treat cells. After the treatment period, 10 *μ*l CCK-8 was added to each well; wells were incubated at 37°C for 1 h and 30 min. The value of OD_450_ was measured by automated microplated reader (Bio-Tek, Winooski, VT, USA). For the clone formation assay, cells were seeded in a 6-well plate at a density of 500 per well and cultured for 48 hours. Then the cells were treated with indicate concentrations of ATO (2*μ*M for MCF-7, 4*μ*M for MDA-MB-231). After one week, cells were fixed with 4% paraformaldehyde and stained with 2% crystal violet. The clones were counted number under a microscope (1 *μ*M = 1*μ*mol/L).

### 2.6. Cell Migration

Wound healing and transwell assays are used to detect the ability of cell migration. For the wound healing assay, cells were seeded in 6-well plates and grown overnight, respectively. Until the cells achieved 80% confluence, they were treated with indicate concentrations of ATO (2*μ*M for MCF-7, 4*μ*M for MDA-MB-231) after a single scratch was made. After continuing to culture 24 h, cells were fixed, stained, and photographed under a light microscope (Olympus, Japan).

For the transwell assay, 20000 cells in 200 *μ*L serum-free medium were added to the upper chamber of transwell insert (Corning, USA), and 600 *μ*L culture medium with 10% FBS or indicate concentrations of ATO was added to the lower chamber. The transwell chamber was incubated for 24 hours. After being fixed and stained, cells in the surface of the lower chamber were observed and recorded under an inverted microscope (Olympus, Japan).

### 2.7. Apoptosis Assay by Flow Cytometry

Flow cytometry was performed to detect apoptosis by annexin V-FITC/PI (Multi Science, China) double staining. MCF-7 and MDA-MD-231 cells in logarithmic growth phase were treated with 2 *μ*M or 4*μ*M ATO for 48 h, DMSO as control group. Then the cells were washed twice with PBS and collected. Following the manufacturer's instructions, cells were stained with annexin V-FITC/PI. The mixtures of cells were detected by flow cytometry (Beckman Coulter, USA).

### 2.8. Reverse Transcription and Quantitative Polymerase Chain Reaction (RT-qPCR)

The mRNA expression level of genes was examined by qPCR (SYBR Green Supermix, Bio-rad, China) normalized to expression of GAPDH. According to the manufacturer's protocol total cellular RNA was extracted using Trizol reagent (Invitrogen, Carlsbad, CA). The cDNA was obtained by RevertAid™ First Strand cDNA Synthesis Kit (Fermentas, Canada). Then the expression level of target genes was analyzed by cDNA. The sequences of the primers for mRNA analysis are listed below: RhoB (Sence: 5'-CGGACTCGCTGGAGAACA-3', Antisense: 5'-GCACCTTGCAGCAGTTGAT-3'); PTEN (Sense: 5'-GGACGAACTGGTGTAATG-3', Antisense: 5'-GCCTCTGACTGGGAATAG-3'); GAPDH (Sense: 5'-CCTTCCTGACAGCCAGTGTG-3', Antisense: 5'-CAGAATGGAAATACTGGAGCAAG-3').

### 2.9. Western Blotting

*C*ells were washed with ice-cold phosphate-buffered saline (PBS) and then lysed in iced RIPA buffer (Beyotime Biotechnology Co., Jiangsu, China). Cell lysate was quantified for protein content after sonication. 20*μ*g of total protein was loaded in each hole, run on 12% SDS-polyacrylamide gel, and transferred to PVDF membranes (Millipore, Billerica, MA). After the transfer, the mixture was blocked with 5% skimmed milk. Membranes were subsequently incubated with primary antibodies, RhoB (dilution 1:1000), p-Akt (dilution 1:500), PTEN (dilution 1:500), E-cadherin(dilution 1:1000), vimentin (dilution 1:1000), snail (dilution 1:1000), *β*-actin (dilution 1:20000), and GAPDH (1:20000), washed with TBST, and incubated with secondary antibodies conjugated with horseradish peroxidase (HRP) (1:10000, Santa Cruz Biotechnology, Santa Cruz, CA). PVDF membranes were scanned using a chemiluminescence imaging system (Tanon, Shanghai, China).

### 2.10. Immunohistochemistry

Immunohistochemistry (IHC) was used to analyze expression of RhoB, PTEN and p-AKT in tumor tissues. The sections were dewaxed in xylene and hydrated in a series of 100, 95, 90, 80, 70, and 60% ethanol solutions. Before incubation with the immunoreagent, endogenous peroxidase activity was inhibited and nonspecific binding of antibody was blocked. The sections were then incubated with primary antibody of RhoB, PTEN, p-AKT (dilution 1:50) overnight at 4°C. The slides were incubated with poly-HRP goat anti-rabbit (Maixin-Bio, Fuzhou, China) for 30 minutes after washing with PBS. The sections were dyed with diaminobenzidine and hematoxylin and then dehydrated in ethanol and cleared in xylene. Images were captured by the Olympus BX40 microscope and CC-12 Soft-Imaging system (Olympus, Tokyo, Japan).

### 2.11. In Vivo Tumor Xenograft Model

Female BALB/c^nu/nu^ nude mice, 28-30 days old and weighing 16-18 g, were maintained under sterile conditions and fed with sterile feed and water. After anesthetizing mice by inhaling isoflurane, 1 × 10^7^ MCF-7 cells in 0.2 ml MEM were injected subcutaneously into the second intramammary gland fat pad of each mouse. Afterwards, tumor-bearing nude mice were fed plus estrogen. When tumors reached around 100-200 mm^3^, mice were randomized into two groups (n=5/group) and treated daily with ATO (10 *μ*g/kg) or DMSO (control) by intraperitoneal injection. The longest surface length (a) of the tumor and its vertical width (b) were measured daily, and the tumor volume was calculated according to 0.5×a×b^2^. When the tumor of control group grew to 1 cm^3^, all mice were sacrificed and tumors were removed and weighed. All operations are carried out following the Guidelines for Animal Experimentation of the Wuhan University. Our protocol was approved by the Ethics Committee for Animal Experimentation and was performed on the basis of the Guidelines for Animal Experimentation of Wuhan University and to the National Institute of Health Guide for the Care and Use of Laboratory Animals.

### 2.12. Statistics

Statistical analysis was performed using GraphPad Prism 7 (Graphpad, USA). Results were expressed as the mean of three independent experiments ± standard deviation (SD) in each figure. For comparison two groups, we used Student's t-test to evaluate the significance test. One-way ANOVA analysis was used to assess statistical significance between three or more groups. *∗p*<0.05 was considered statistically significant.

## 3. Results

### 3.1. ATO Inhibits Cell Proliferation and Promotes Cell Apoptosis in Breast Cancer Cells

**Figure 1:**
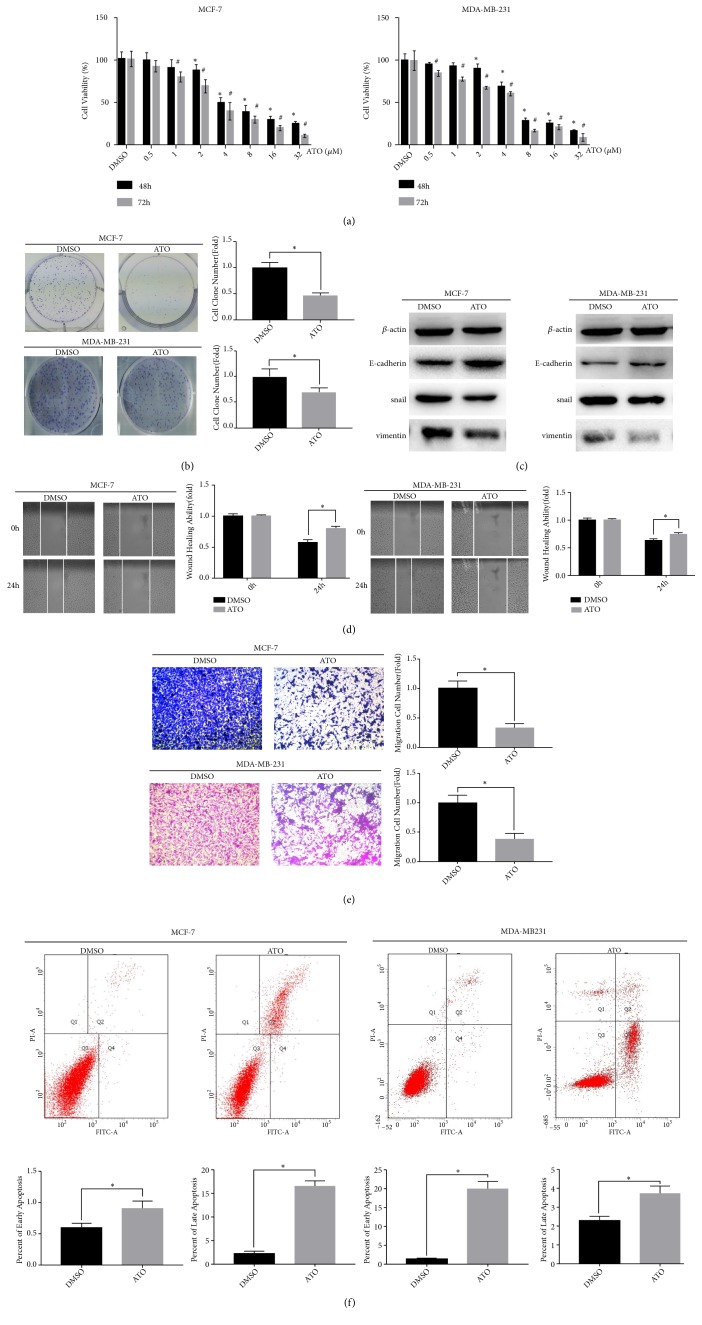
Atorvastatin inhibits proliferation, migration, and EMT of breast cancer cells, while promoting cell apoptosis. MDA-MB-231 cells were treated with 4*μ*M ATO and MCF-7 cells were treated with 2 *μ*M ATO in (b), (c), (d), (e), and (f). (a) ATO inhibits the proliferation of breast cancer cells determined by CCK-8 assay. (b) ATO suppresses the proliferation of breast cancer cells determined by the clone formation assay. (c) After being treated with ATO for 48 h, the corresponding proteins of EMT were evaluated in breast cancer cells by Western blotting. (d) Wound healing assay reveals that ATO reduces the ability of migration of breast cancer cells. (e) ATO suppresses the invasion of breast cancer cells which were revealed by transwell assay. (f) ATO induces early apoptosis and late apoptosis of breast cancer cells which was detected by annexin V-FITC and PI staining. Data were presented as mean ± SD from three independent measurements. *∗p* < 0.05.

In order to examine the biological effects of ATO in breast cancer cells, MCF-7 and MDA-MB-231 cells were incubated with different concentrations of ATO (0.5*μ*M-32*μ*M) for 48 and 72 h. Then we detected cells viability using CCK8; the results showed that ATO inhibited the cells proliferation in a time- and concentration-dependent manner (Figure 1(a)). At the same time, we founded that MCF-7 cells were more sensitive to the treatment of ATO than MDA-MB-231 cells. Based on the results of the CCK8 assay, we treated MCF-7 cells with 2 *μ*M ATO and treated MDA-MB-231 cells with 4 *μ*M ATO in subsequent experiments. Clone formation assays revealed that ATO significantly inhibited the ability of clonal formation of breast cancer cells (Figure 1(b)). Apoptosis is one of the important factors affecting cell proliferation. Our results suggested that the numbers of early apoptosis and late apoptosis of breast cancer cells were increased significantly after 48 h of treatment with ATO (Figure 1(f)).

### 3.2. ATO Inhibits Cell Invasion and Regulates the Expression of EMT-Related Proteins in Breast Cancer Cells

Subsequently, we used the transwell and wound healing assays to detect cell invasion and migration of breast cancer cells. The results of wound healing assays showed that ATO significantly inhibited the two-dimensional invasion ability of MDA-MB-231 cells and MCF-7 cells (Figure 1(d)). The results of transwell indicated that ATO significantly inhibited the three-dimensional invasion ability of breast cancer cells (Figure 1(e)). Next, we used Western blot to detect the expression of EMT-related proteins including E-cadherin, vimentin, and snail. The results showed that the expression levels of E-cadherin were upregulated, while vimentin and snail were downregulated after being treated with ATO in MCF-7 and MDA-MB-231 cells (Figure 1(c)).

### 3.3. The mRNA and Protein Expression Level of RhoB Was Upregulated in Statin-Treated Breast Cancer Cells

**Figure 2:**
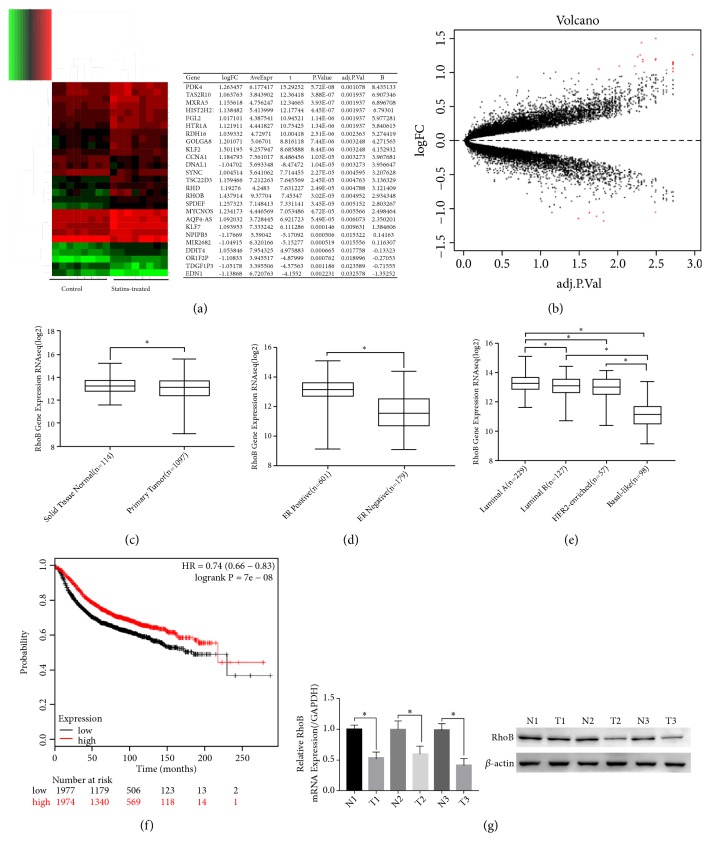
RhoB is significantly downregulated in human breast cancer tissues. (a-b) Transcriptome profiling data from MDA-MB-231 treated with Fluvastatin or mock-treated control cells was downloaded from GSE33552. (a) Heat map and list of the differentially expressed genes of statins-treated cells and mock-treated cells (log⁡FC > 1). (b) Volcano map of the differentially expressed genes of the two groups (log⁡FC > 1). (c-e) RhoB expression in breast cancer patients. Data was downloaded from TCGA and determined using the Metabolic gEne RApid Visualizer. (c) RhoB is significantly downregulated in primary tumor comparing with normal tissue. (d) Compared with ER-negative patients, the RhoB mRNA expression levels of ER-positive patients were significantly increased. (e) The expression of RhoB was lower in luminal B, HER2-enriched and basal-like subtypes when compared with luminal A and basal-like subtype being the lowest. (f) Kaplan-Meier relapse-free survival (RFS) curves of RhoB (n = 3951, P = 7E-06 by log-rank test for significance). Data were analyzed using the Kaplan-Meier Plotter. (g) RhoB mRNA and protein expression were downregulated in breast cancer tissues than paired normal breast tissues which was analyzed by RT-qPCR and Western blotting. Values represent the mean ± SD from three independent measurements. *∗p* < 0.05.

**Figure 3:**
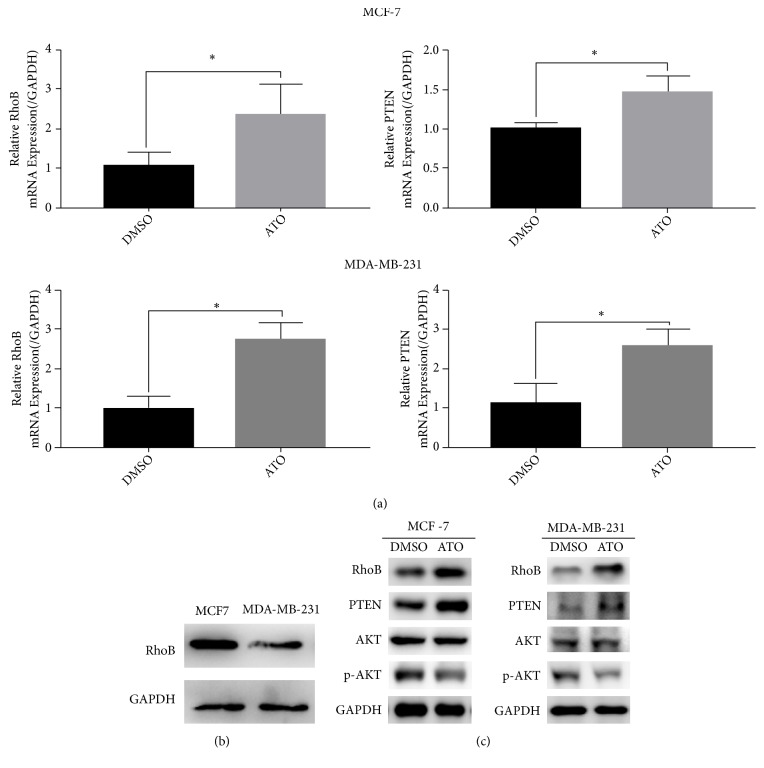
Atorvastatin upregulates the expression of RhoB and activates the PTEN/AKT pathway. MDA-MB-231 cells were treated with 4*μ*M ATO and MCF-7 cells were treated with 2 *μ*M ATO for 48 h. (a) RhoB and PTEN mRNA expression were upregulated after the treatment of ATO which was analyzed by RT-qPCR. (b) RhoB protein expression was detected by Western blotting in breast cancer cell lines MCF-7 and MDA-MB-231. (c) The effect of ATO on the protein levels of RhoB, PTEN, p-AKT, and AKT. Data were presented as mean ± SD from three independent measurements. *∗p* < 0.05.

To screen for potential targets for atorvastatin in breast cancer cells, we analyzed difference in transcriptome levels of MDA-MB-231 cells which were treated with lovastatin from GSE33552 dataset, and then we selected the genes with significant differences in expression levels after statin treatment of MDA-MB-231 cells. The genes include 15 significantly upregulated genes and 6 significantly downregulated genes (Fold Change>10), including RhoB (Figures 2(b) and 2(c)). Subsequently, we used RT-qPCR and Western blot to detect the expression levels of RhoB mRNA and protein in MDA-MB-231 cells and MCF-7 cells after ATO treatment. The results showed that ATO upregulated RhoB mRNA and protein expression in breast cancer cells (Figures 3(a) and 3(c)).

### 3.4. The Expression Level of RhoB Is Downregulated in Cancer Tissues and Low Expression of RhoB Is Associated with Poor Prognosis in Breast Cancer Patients

To assess the expression of RhoB in breast cancer and normal tissues, we analyzed the expression levels and related clinical data of RhoB in tissues derived from the TCGA database using the Metabolic gEne RApid Visualizer online tool. Compared with normal breast tissues, RhoB expressions were lower in breast cancer tissues (Figure 2(c)). To verify this result, we used RT-qPCR and Western blot to detect 3 pairs of breast cancer and corresponding adjacent tissues. The results showed that the mRNA and protein expression levels of RhoB in breast cancer tissues were lower than those in adjacent tissues (Figure 2(g)). Furthermore, combined with clinical data, we found that RhoB expression levels were significantly higher in estrogen receptor-positive breast cancer tissues than in estrogen receptor-negative tissues (Figure 2(d)). Subsequently we compared the differences of RhoB expression levels in breast cancer tissues with different PAM50 types. Among the four types, the expression level of RhoB was the highest in luminal A breast cancer tissues and the expression level of RhoB was the lowest in basal-like breast cancer tissues (Figure 2(e)). The difference is significant. We then used online database Kaplan-Meier Plotter to evaluate the expression levels of RhoB of relapse-free survival (RFS) of breast cancer patients (Figure 2(f)). The database classified breast cancer patients into high expression group (red line) and low expression group (black line) based on the median RhoB expression level. By comparing the difference in RFS between the two groups, we found that patients with high RhoB expression displayed significantly longer RFS (HR = 0.74[0.66–0.83],* p* = 7e^−8^) than those with lower RhoB expression.

### 3.5. RhoB Inhibits the Proliferation, Invasion, and EMT in Breast Cancer Cells

To explore the role of RhoB in breast cancer, we knocked down RhoB in MCF-7 cells (Figure 5(a)) and overexpressed RhoB in MDA-MB-231 cells (Figure 6(a)) in accordance with RhoB protein levels in MCF-7 cells and MDA-MB-231 cells, followed by detection of cell biological effects. The CCK8 assay and clone formation assays were used to examine the effect of RhoB on cell proliferation. The results showed that overexpression of RhoB significantly inhibited the cell proliferation (Figures 6(b) and 6(c)), while knockdown of RhoB was significantly enhanced it(Figures 5(b) and 5(c)). Subsequently, we used the wound healing and transwell assay to examine the effect of RhoB on cell invasion. The results showed that overexpression of RhoB inhibited the invasive ability of MDA-MB-231 cells (Figures 6(d) and 6(e)), while knocking down RhoB promoted the two- the invasive ability of MCF-7 cells (Figures 5(d) and 5(e)). Further, we used Western blot to detect the expression levels of related proteins in the EMT process. The results of Western blot indicated that the expression of E-cadherin increased in MDA-MB-231 cells (Figure 6(f)), and the expression levels of vimentin and snail decreased after overexpression of RhoB. After knockdown of RhoB, the expression of E-cadherin in MCF-7 cells decreased and the expression levels of vimentin and snail increased (Figure 5(f)).

### 3.6. ATO Inhibits the PTEN/AKT Signaling Pathway in Breast Cancer Cells via Upregulating the Expression of RhoB

It is well known that activation of the PTEN/AKT signaling pathway can promote the proliferation, invasion, and EMT of breast cancer cells. Therefore, we speculated that ATO may suppress breast cancer cells by inhibiting the PTEN/AKT signaling pathway via upregulating RhoB. In breast cancer cells treated with atorvastatin, RT-qRCR was used to analyze the mRNA expression level of RhoB and PTEN, and Western blot was used to detect the protein expression level of RhoB or PTEN/AKT signaling pathway-related molecules-PTEN, AKT, and p-AKT. The results showed that, after treatment with ATO in breast cancer cells, the mRNA and protein levels of RhoB and PTEN were significantly upregulated, while the protein levels of p-AKT were significantly downregulated (Figures 3(a) and 3(c)). To detect the effect of RhoB on PTEN/AKT signaling pathway in breast cancer cells, we respectively used Western blot to detect changes in the expression levels of PTEN/AKT signaling pathway-related protein AKT, p-AKT, and PTEN after overexpression and knockdown of RhoB in MDA-MB-231 cells and MCF-7. The results showed that, after overexpression of RhoB, the protein level of PTEN in MDA-MB-231 cells increased and the protein level of p-AKT decreased (Figure 6(f)). After knockdown of RhoB, the protein level of PTEN in MCF-7 cells decreased and the protein level of p-AKT increased (Figure 5(f)). The above experimental results indicate that RhoB can inhibit the PTEN/AKT signaling pathway in breast cancer cells.

### 3.7. ATO Inhibits the Tumor Growth and Upregulates RhoB in Nude Mice

**Figure 4:**
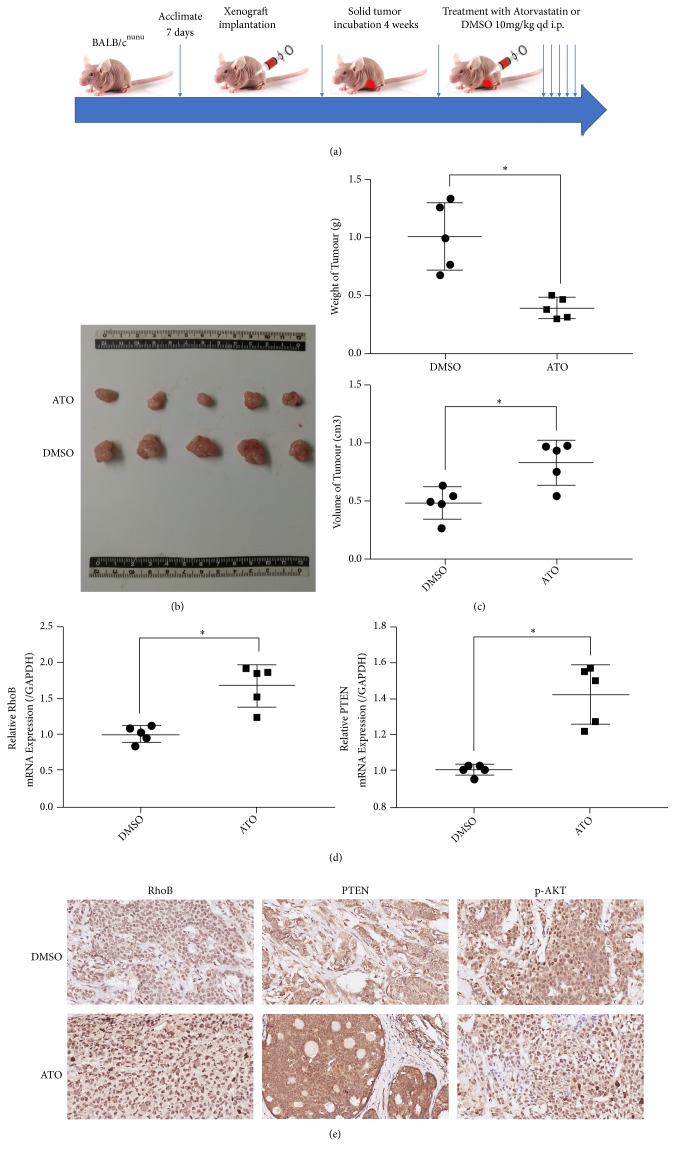
Atorvastatin inhibits growth of tumor in the MCF-7 xenograft model. (a) Experimental design of MCF-7 xenograft model. (b) Atorvastatin inhibits tumor growth of xenografted mice. (c) Tumor weight and volume from xenografted mouse. (d) RhoB and PTEN mRNA expression in tumor tissue were upregulated after the treatment of atorvastatin which was analyzed by RT-qPCR. (e) The protein levels of RhoB and PTEN were higher in cancer tissues from atorvastatin-treated mouse while the protein levels of p-AKT were lower. Values represent the mean ± SD from three independent measurements. *∗p* < 0.05.

**Figure 5:**
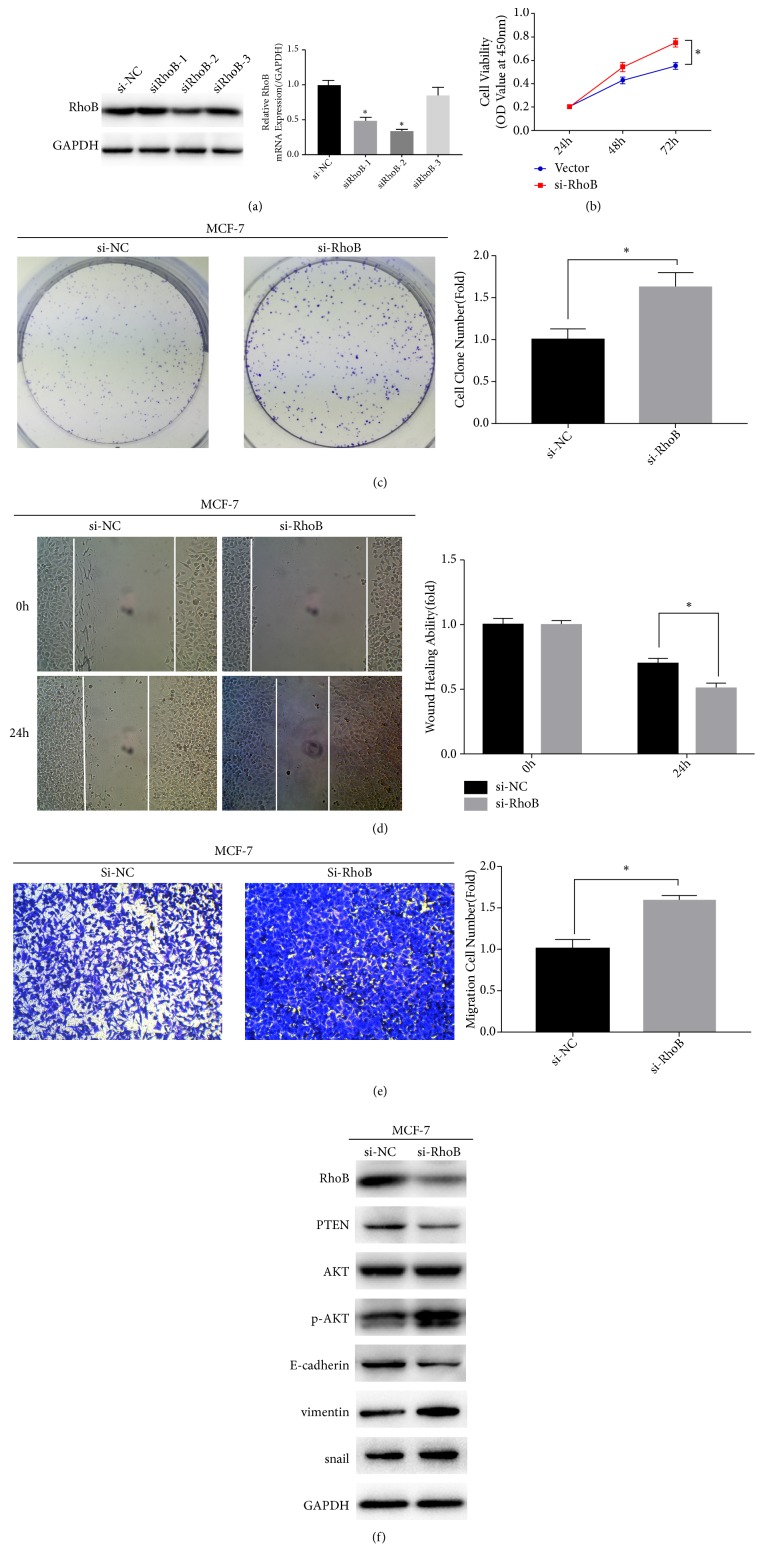
Knockdown of RhoB promotes MCF-7 cells migration, proliferation, and EMT and upregulates PTEN/AKT pathway. (a) MCF-7 cells were transfected with small interfering RNA of RhoB (siRhoB1,2,3) or negative control (si-NC) and detected by RT-qPCR and Western blotting. Si-RhoB2 was chosen for the further experiment. (b) Knockdown of RhoB enhances the proliferation of MCF-7 cells detected by the CCK-8 assay. (c) RhoB knockdown upregulates the proliferation of MCF-7 cells detected by the colon formation assay. (d) Wound healing assay reveals that RhoB knockdown enhances the ability of migration of MCF-7 cells. (e) RhoB knockdown enhances the migration ability of MCF-7 cells revealed by transwell assay. (f) The effect of transfecting with si-RhoB or si-NC on the protein levels of RhoB, PTEN, p-AKT, AKT, E-cadherin, vimentin, and snail in MCF-7 cells. Values represent the mean ± SD from three independent measurements. *∗p* < 0.05.

**Figure 6:**
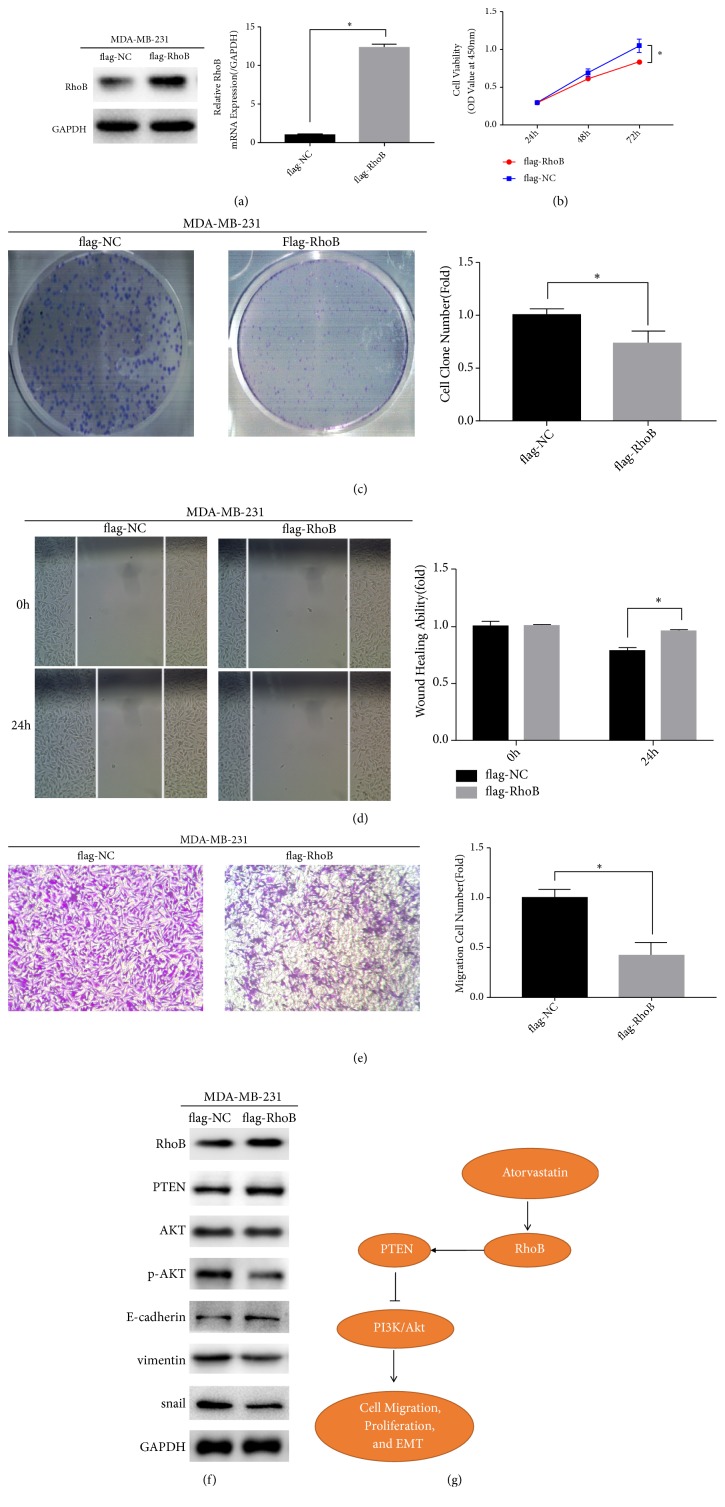
Overexpression of RhoB inhibits MDA-MB-231 cells migration, proliferation, and EMT and downregulates PTEN/AKT pathway. (a) MDA-MB-231 cells were transfected with flag-PAK7 overexpression plasmid or flag-NC negative control vector and detected by RT- qPCR and Western blotting. (b) Overexpression of RhoB inhibits the proliferation of MDA-MB-231 cells which was detected by the CCK-8 assay. (c) RhoB overexpression downregulates the proliferation of MDA-MB-231 cells detected by the colon formation assay. (d) Wound healing assay reveals that RhoB overexpression reduces the ability of migration of MDA-MB-231 cells. (e) RhoB overexpression suppresses the migration of MDA-MB-231 cells revealed by transwell assay. (f) The effect of transfecting with flag-RhoB overexpression plasmid or flag-NC negative control vector on the protein levels of RhoB, PTEN, p-AKT, AKT, E-cadherin, vimentin, and snail in MDA-MB-231 cells. (g) Working model for the regulation of PTEN/AKT pathway suppressed by atorvastatin via upregulating the expression of RhoB. Values represent the mean ± SD from three independent measurements. *∗p* < 0.05.

In order to detect the effect of atorvastatin on the growth of breast cancer in the body, MCF-7 cells were injected subcutaneously into the second breast mats of 4-5 weeks old BALB / C ^nu  /  nu^ nonthymic mice. When the tumor volume reached 100-200mm^3^, mice were divided into two groups and treated with DMSO or ATO (10mg/kg) every day by intraperitoneal injection (Figure 4(a)). The results revealed that the mice injected with ATO showed a decrease in tumor volume and tumor weight, compared with the DMSO injection group (Figures 4(b) and 4(c)), which suggested that ATO can inhibit the growth of breast cancer* in vivo*. Subsequently, the mRNA and protein expression level of RhoB, detected by RT-qPCR and Western blot, in the tumor tissues treated with ATO was higher than that treated with DMSO (Figures 4(d) and 4(e)). Subsequently, RT-qPCR and IHC were used to detect the changes of relevant indicators in tumor tissues of xenografted mice treated with ATO. The results showed that, compared with the DMSO treatment group, the mRNA and protein expression levels of PTEN and RhoB increased significantly in the tumor tissues of mice treated with atorvastatin, while the protein levels of p-AKT decreased significantly.

## 4. Discussion

ATO is widely used for treatment of cardiovascular disease. In recent years, many studies have demonstrated its antitumor effect. Retrospective studies have indicated that the use of statins can reduce tumor-related mortality [9]. For example, neoadjuvant chemoradiotherapy combined with statins for rectal cancer reduced the pathological grade of patients [21]. In vitro, statins induce apoptosis in human colon cancer cells and prostate cancer cells [21]. Simvastatin inhibits breast cancer cell proliferation via inactivating MAPK/ERK signaling pathways [22]. As for ATO in breast cancer, ATO promotes autophagy and apoptosis in breast cancer cells [23, 24]. In this study we analyzed the effect of ATO using low invasive luminal breast cancer cell line MCF-7 and highly invasive basal-like breast cancer cell line MDA-MB-231. Our study shows that ATO significantly inhibits the proliferation and invasion and promotes cell apoptosis of MCF-7 and MDA-MB-231 cells. ATO also inhibits EMT of breast cancer cells by regulating the expression of EMT-related proteins. Furthermore, atorvastatin significantly inhibits tumor growth in a tumor-bearing model constructed using MCF-7 cells. Our experimental results further confirm the inhibitory effect of ATO on breast cancer. But the mechanism by which ATO inhibits breast cancer cells remains to be explored.

In a phase II clinical trial, researchers found that RhoB expression increased in tissue samples from breast cancer patients after treatment of ATO [10]. We further validated this view by analyzing the changes in the transcriptional level of MDA-MB-231 cells after being treated by lovastatin. Subsequently, we examined changes in RhoB protein and mRNA levels in breast cancer cells and animal breast tumor tissues after atorvastatin treatment. The results of RT-qPCR and Western blot showed that ATO promoted RhoB expression. RhoB mediates the regulation of various cell biological functions, including cytoskeletal growth, signal identifying, cytosport, apoptosis, neural crest migration, cell motility, and membrane trafficking and has a certain relationship with tumor growth and proliferation [25]. By analyzing the data from TCGA database, we found that RhoB expression was significantly downregulated in breast cancer tissues. Our RT-qPCR and Western blot results also support and confirm the finding. Studies have reported that the expression of RhoB is positively correlated with the expression of estrogen receptor, which is supported by our result [26]. In vitro, studies have reported that RhoB, which is low-expressed in gastric cancer, lung cancer, ovarian cancer, and thyroid cancer cell lines, inhibits tumor cell proliferation, migration, and invasion [27–30]. But the function of RhoB in breast cancer is still unknown. Verification in human breast cancer cell lines demonstrates that there is a signal pathway crossover between estrogen receptor alpha and RhoB [26]. In the present study, we analyzed the data in the database and found that, in different PAM50 subtype of breast cancer, RhoB mRNA expression was highest in the subtype with lowest malignancy (luminal A), while RhoB mRNA was the lowest in the subtype with highest malignancy (basal-like). Survival analysis revealed that patients in the RhoB mRNA high expression group had a longer overall survival time than in the lower expression group. All in all, these findings provide evidence that RhoB acts as a tumor suppressor gene. However, the mechanism of RhoB inhibition of breast cancer remains to be studied.

The PTEN/AKT signaling pathway is involved in the regulation of multiple cellular dysfunctions in breast cancer cells, including proliferation, metabolism, and genomic instability [31]. RhoB plays an important role in the PI3K/AKT pathway, and studies have shown that RhoB mediates regulation of the PI3K/AKT pathway in gastric cancer cells, inhibiting invasion and migration by reducing the expression level of p-AKT [32, 33]. Therefore, we hypothesize that atorvastatin may inhibit tumorigenesis by suppressing the PTEN/AKT pathway via upregulating the expression of RhoB in breast cancer. Our findings showed that, in breast cancer cells and animal tumor tissues treated with ATO, PTEN protein levels were elevated and p-AKT protein levels were decreased, indicating that the PTEN/AKT pathway was inhibited. Based on the protein levels of RhoB in MCF-7 cells and MDA-MB-231 cells, we overexpressed RhoB in MDA-MB-231 cells and knocked out RhoB in MCF-7 cells. Subsequent experiments showed that RhoB significantly inhibited the proliferation, invasion and EMT of breast cancer cells, confirming that RhoB plays a role in tumor suppressor function in breast cancer cells. We then observed that, after overexpression of RhoB, the PTEN/AKT signaling pathway was inhibited, and the signaling pathway was activated after knockdown of RhoB. Our study confirms that RhoB inhibits breast cancer proliferation, invasion, and EMT by inhibiting PTEN/AKT signaling pathway. However, the specific mechanism between RhoB and PTEN/AKT signaling pathway remains to be further explored.

In summary, ATO inhibits the expression level of p-AKT by positively regulating the expression level of RhoB and increases the expression level of PTEN, thereby inhibiting the PI3K/AKT pathway and exerting its inhibitory effect on breast cancer. This study further supports the important role of ATO in the adjuvant treatment of breast cancer and suggests that RhoB has the potential to become a new biomarker for breast cancer.

## Data Availability

The data used to support the findings of this study are available from the corresponding author upon request.
